# Aggravated myocardial infarction-induced cardiac remodeling and heart failure in histamine-deficient mice

**DOI:** 10.1038/srep44007

**Published:** 2017-03-08

**Authors:** Jinmiao Chen, Tao Hong, Suling Ding, Long Deng, Mieradilijiang Abudupataer, Weiwei Zhang, Minghong Tong, Jianguo Jia, Hui Gong, Yunzeng Zou, Timothy C. Wang, Junbo Ge, Xiangdong Yang

**Affiliations:** 1 Shanghai Institute of Cardiovascular Diseases, Zhongshan Hospital, and Institutes of Biomedical Sciences, Fudan University, Shanghai, 200032, China; 2 Department of Cardiac Surgery, Zhongshan Hospital, Fudan University, Shanghai, 200032, China; 3 Department of Clinical Medicine, TongRen Hospital Affiliated with Shanghai Jiao Tong University, Shanghai, 200000, China; 4 Department of Medicine and Irving Cancer Research Center, Columbia University, New York, NY 10032, USA

## Abstract

Histamine has pleiotropic pathophysiological effects, but its role in myocardial infarction (MI)-induced cardiac remodeling remains unclear. Histidine decarboxylase (HDC) is the main enzyme involved in histamine production. Here, we clarified the roles of HDC-expressing cells and histamine in heart failure post-MI using HDC-EGFP transgenic mice and HDC-knockout (HDC^−/−^) mice. HDC^+^CD11b^+^ myeloid cell numbers markedly increased in the injured hearts, and histamine levels were up-regulated in the circulation post-MI. HDC^−/−^ mice exhibited more adverse cardiac remodeling, poorer left ventricular function and higher mortality by increasing cardiac fibrogenesis post-MI. *In vitro* assays further confirmed that histamine inhibited heart fibroblast proliferation. Furthermore, histamine enhanced the signal transducer and activator of transcription (STAT)-6 phosphorylation level in murine heart fibroblasts, and the inhibitive effects of histamine on fibroblast proliferation could be blocked by JAK3/STAT6 signaling selective antagonist. STAT6-knockout (STAT6^−/−^) mice had a phenotype similar to that of HDC^−/−^ mice post-MI; however, in contrast to HDC^−/−^ mice, the beneficial effects of exogenous histamine injections were abrogated in STAT6^−/−^ mice. These data suggest that histamine exerts protective effects by modulating cardiac fibrosis and remodeling post-MI, in part through the STAT6-dependent signaling pathway.

Despite advances in medical and interventional therapy for myocardial infarction (MI), many post-MI patients still develop into heart failure^1^,^2^. Progression from MI to heart failure is closely associated with cardiac remodeling with respect to both geometry and function. The numbers of current therapeutic strategies intended to prevent MI-induced cardiac remodeling and heart failure remain limited because the mechanism underlying this phenomenon is unclear. Thus, there remains a great need to identify novel targets to prevent adverse cardiac remodeling after MI^3^. MI severity is the most critical determinant of cardiac remodeling; however, numerous cellular events, including cardiomyocyte apoptosis, continuous or chronic inflammatory responses, cardiomyocyte hypertrophy, and interstitial fibrosis, can also influence disease evolution.

Histamine is a biogenic amine involved in a variety of physiological and pathophysiological functions in the context of allergic responses, gastric acid secretion, immune modulation, and carcinogenesis^4^,^5^,^6^,^7^. Although increases in histamine levels during the early stage of MI have been documented^8^,^9^,^10^, the origin and effects of histamine with respect to the development of heart failure post-MI, as well as the mechanisms underlying these effects, are still controversial. Mast cells have been known to store and secrete histamine; however, histidine decarboxylase (HDC), the main enzyme responsible for generating endogenous histamine, is primarily expressed in CD11b^+^Gr1^+^ myeloid cells rather than in mast cells^7^. Approximately 90% of CD11b^+^Gr1^+^ myeloid cells residing in the bone marrow highly express HDC and are thought to represent immature myeloid cells^7^. A recent study by Deng *et al*. demonstrated that a large number of HDC-expressing CD11b^+^Gr1^+^ myeloid cells infiltrated into the infarcted hearts and that HDC^+^ myeloid cell-derived histamine could protect cardiac function by reducing cardiomyocyte apoptosis in the early stages of MI^10^. However, the roles of HDC-expressing CD11b^+^ myeloid cells and histamine in MI-induced heart failure have not yet been elucidated.

There are four known histamine receptors. Of these, histamine receptors 1 and 2 are highly expressed in the heart. However, the signaling pathways downstream of these G-protein-coupled receptors with respect to cardiac remodeling have not been well studied^11^. Histamine has previously been linked to STAT1 and STAT4 phosphorylation^12^,^13^. Additionally, STAT6 activation regulates Th2 differentiation, and histamine also affects Th1/Th2 balance^14^. As our previous study showed that STAT6 mRNA expression levels were significantly down-regulated in the CD11b^+^Gr1^+^ myeloid cells of HDC^−/−^ mice compared with those of WT mice^7^, whether the STAT6 signaling pathway is a downstream target of histamine in the setting of cardiac fibrogenesis and remodeling requires further investigation.

In this study, we showed that genetic deficiency of histamine promoted cardiac fibrogenesis and dampened heart function post-MI. Furthermore, we showed that the STAT6 signaling pathway appeared to be involved in the critical functions of histamine post-MI.

## Results

### Histamine and HDC-expressing myeloid cell numbers increased in the circulation and heart during cardiac remodeling post-MI

**Figure 1:**
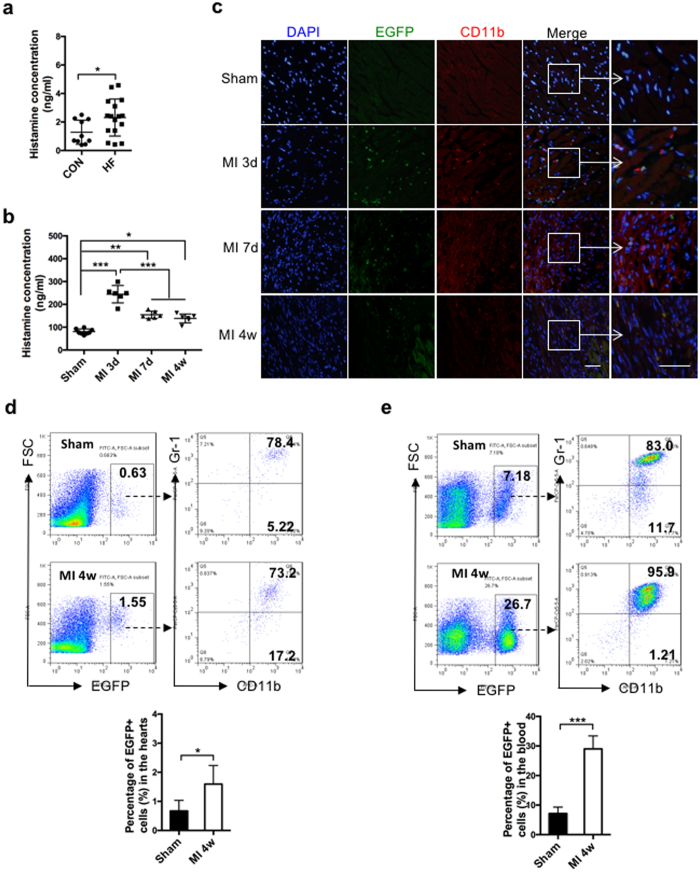
Histamine expression and HDC-expressing CD11b^+^ myeloid cells in patients and a murine model with heart failure. (**a**) The serum histamine levels in heart failure patients and healthy control subjects (Control: 1.22 ± 0.82 ng/ml, heart failure: 2.39 ± 1.30 ng/ml; n = 10–16). (**b**) The higher serum histamine levels in MI mice than the sham group (Sham: 80.51 ± 11.77 ng/ml, MI 3d: 243.80 ± 38.03 ng/ml, MI 7d: 155.70 ± 15.98 ng/ml, MI 4w: 137.5 ± 19.42 ng/ml; n = 6–8). (**c**) Representative images of infarcted hearts showed that a large amount of HDC-expressing CD11b^+^ myeloid cells infiltrated in the hearts of HDC-EGFP mice at MI 3d, MI 7d, and MI 4w compared with the sham group (scale bar = 50 μm; n = 3–5). (**d**,**e**) The percentages of EGFP^+^ cells were analyzed by FACS in the infarcted hearts (**d**) and peripheral blood (**e**) of mice at 4 weeks post-MI. FSC indicates Forward Scattering. (n = 6–8). *p < 0.05, **p < 0.01, ***p < 0.001.

To investigate the roles of histamine and HDC-expressing myeloid cells in cardiac remodeling, we first examined histamine levels in the circulation of heart failure patients and healthy control subjects. The results of this examination showed that serum histamine concentrations significantly increased in heart failure patients (Fig. 1a). We established an MI-induced cardiac remodeling mouse model by permanently ligating the left anterior descending (LAD) coronary artery. We then detected serum histamine levels in wild-type (Balb/C background, WT) mice at 3 days, 7 days, and 4 weeks after MI. The serum histamine levels in MI mice significantly increased on day 3 after MI compared with control mice that underwent a sham surgical operation. Higher serum histamine levels were still detected in heart failure mice compared with control mice at 4 weeks post-MI (Fig. 1b). Given that HDC is the main enzyme responsible for endogenous histamine production, we employed HDC-EGFP bacterial artificial chromosome (BAC) transgenic mice (HDC-EGFP mice, C57BL/6 background) to track HDC-expressing cells during the processes of cardiac healing and remodeling. The immunofluorescence staining data showed that EGFP^+^ cells were rare in the hearts of sham mice. In contrast, the numbers of EGFP^+^ cells dramatically increased in the border zone of the infarcted hearts, reaching a peak on day 3 after MI. The numbers of EGFP^+^ cells in the infarcted hearts during the repair and remodeling period (7 days - 4 weeks post-MI) decreased but remained higher than sham controls (Fig. 1c). The results of the immunofluorescence co-staining experiments involving anti-CD11b demonstrated that the majority of EGFP^+^ cells in the infarcted hearts of HDC-EGFP mice were CD11b positive (Fig. 1c). The FACS data demonstrated that the percentage of EGFP^+^ cells significantly increased in the infarcted hearts and blood of HDC-EGFP mice at 4 weeks after MI. These data also demonstrated that EGFP^+^ cells were largely confined to CD11b^+^ myeloid cells, particularly CD11b^+^Gr-1^+^ myeloid cells (Fig. 1d,e). In addition, we found that EGFP^+^CD11b^+^ cell numbers markedly increased in the bone marrow and spleens of HDC-EGFP mice on day 3 post-MI (Supplementary Fig. S1a,b), suggesting that cardiac injury not only promoted the release of EGFP^+^ myeloid cells into the circulation but also led to the production of EGFP^+^CD11b^+^ myeloid cells in the reservoirs.

### Histamine deficiency aggravated cardiac remodeling and dysfunction post-MI

**Figure 2:**
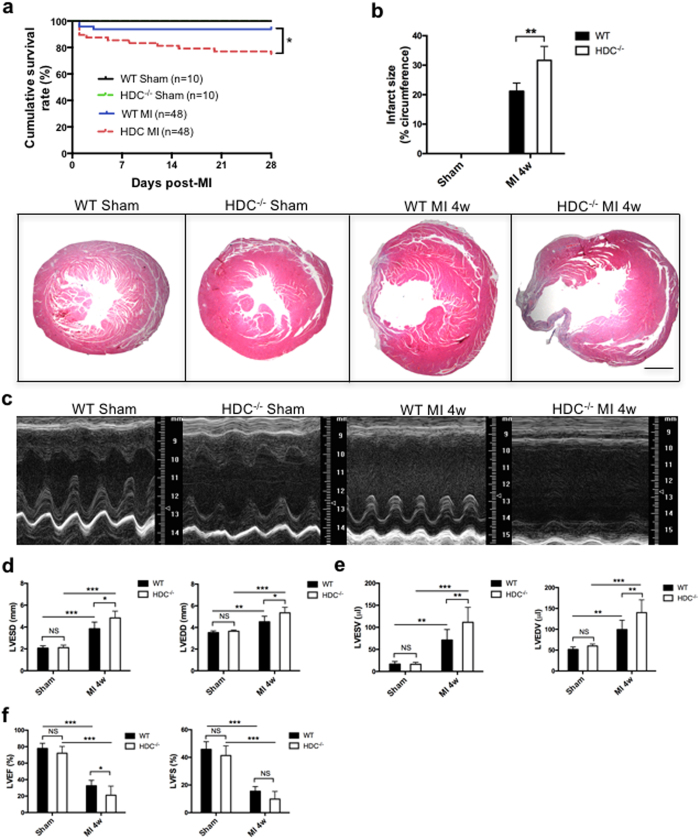
Histamine deficiency aggravated cardiac remodeling and dysfunction post-MI. (**a**) The cumulative survival rate of HDC^−/−^ and WT mice post-MI. (**b**) H&E staining showed that histamine deficiency caused larger infarct size in HDC^−/−^ mice than WT mice at 4 weeks post-MI (scale bar = 500 μm; n = 8–10). (**c**) Representative echocardiographic images. (**d–f**) More serious dilative cardiac remodeling and worse cardiac function in HDC^−/−^ mice than WT mice post-MI (n = 8–10). NS, not statistically significant, *p < 0.05, **p < 0.01, ***p < 0.001.

To elucidate the effects of histamine on MI-induced cardiac remodeling, we established a murine MI model in both HDC^−/−^ mice (Balb/C background) and WT mice. At baseline, HDC^−/−^ and WT mice (8 weeks old) showed no significant differences in both cardiac geometry and function (Table 1). Four weeks after MI, we noted significantly lower cumulative survival rate in HDC^−/−^ mice than in WT control mice (Fig. 2a). Furthermore, HDC^−/−^ mice exhibited worse pathological cardiac remodeling and heart failure than WT control mice. The H&E staining results demonstrated that histamine deficiency caused larger infarcted sizes in HDC^−/−^ mice than WT mice at 4weeks after MI (Fig. 2b). The results of the two-dimensional, high-resolution echocardiographic assay showed worse cardiac dysfunction in HDC^−/−^ mice than WT mice, as the former group of mice exhibited significant decrease in left ventricular ejection fraction (LVEF) and significant increase in left ventricular end-systolic diameter (LVESD), left ventricular end-diastolic diameter (LVEDD), left ventricular end-systolic volume (LVESV) and left ventricular end-diastolic volume (LVEDV) compared with the latter group of mice (Fig. 2c–f). In addition, we found that lung weights were higher in HDC^−/−^ mice than WT control mice at 4 weeks after MI, a finding suggestive of the presence of worse pulmonary edema in HDC^−/−^ mice than WT mice during the decompensated phase of heart failure (Supplementary Fig. S2a,b). Taken together, these data suggested that endogenous histamine had beneficial effects in cardiac healing and remodeling post-MI.

### Cardiac fibrosis was enhanced in the infarcted hearts of HDC^−/−^ mice

**Figure 3:**
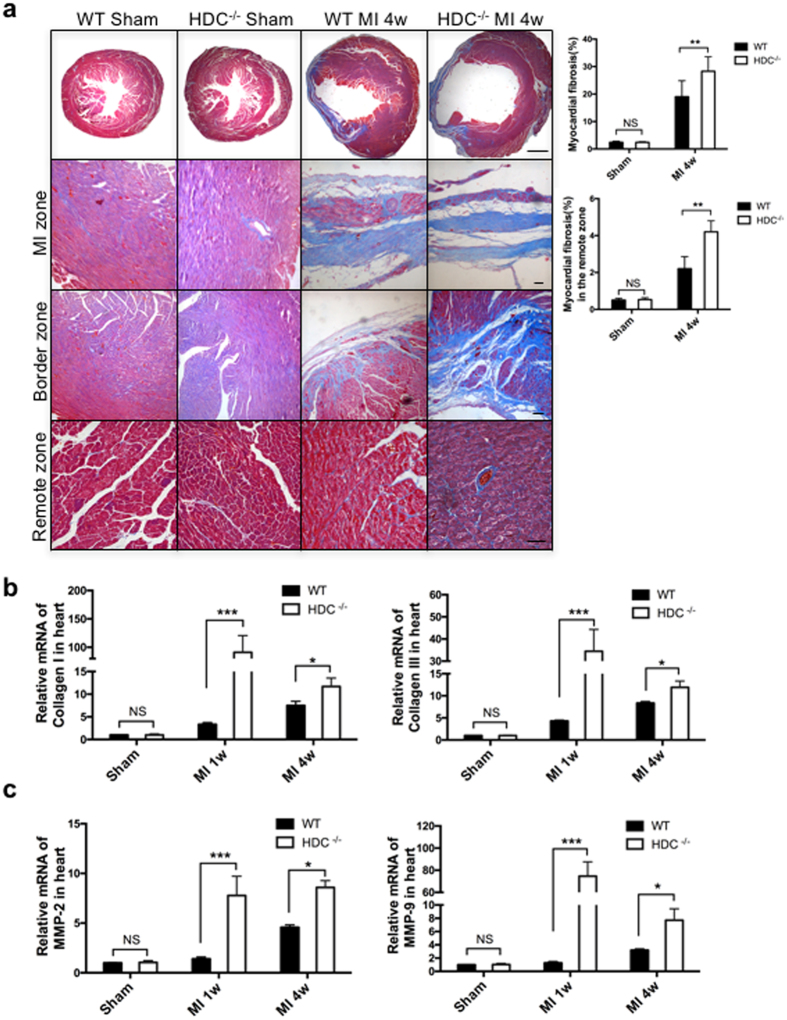
Histamine deficiency increased fibrosis in the infarcted heart. (**a**) Masson trichrome staining showed that fibrosis significantly increased in HDC^−/−^ mice post-MI (the top scale bar = 500μm and other scale bars = 50 μm; n = 8–10). (**b**) Collagen I and III mRNA expression levels in the infarcted heart of HDC^−/−^ mice and WT controls were measured by qRT-PCR (n = 3). (**c**) MMP-2 and MMP-9 mRNA expression levels in the infarcted hearts of HDC^−/−^ mice and WT controls were measured by qRT-PCR (n = 3). NS, not statistically significant, *p < 0.05, **p < 0.01, ***p < 0.001.

A previous study demonstrated that cardiomyocyte apoptosis in the infarcted hearts markedly increased in HDC^−/−^ mice than WT mice during the early stages of MI^10^. However, in this study we observed that there was no significant difference in the degree of cardiomyocyte apoptosis in HDC^−/−^ mice compared with WT mice at 4 weeks post-MI (Supplementary Fig. S3a). It seemed that endogenous histamine may have other ways of protecting cardiac function during the remodeling stage. In the sham groups, the Masson trichrome staining and Picro-Sirius Red staining results showed that there was no significant difference in cardiac fibrosis between HDC^−/−^ and WT mice. However, cardiac fibrosis was significantly enhanced in HDC^−/−^ mice compared with WT control mice at 4 weeks post-MI (Fig. 3a and Supplementary Fig. S3b). Consistent with these findings, the qRT-PCR data confirmed that collagen I and III mRNA expression levels in the infarcted hearts were significantly up-regulated in HDC^−/−^ mice compared with WT mice post-MI, particularly at the 1-week time point, known as the post-MI fibroblast proliferation stage (Fig. 3b). The expression levels of collagen metabolism markers, such as carboxyterminal propeptide of type I procollagen (PICP) and aminoterminal propeptide of type III procollagen (PIIINP), also increased in the serum of HDC^−/−^ mice compared with controls at 1week and 4 weeks post-MI (Supplementary Fig. S3c). Furthermore, the qRT-PCR results showed that MMP-2 and MMP-9 mRNA expression levels, which may contribute to post-MI cardiac dilation, were significantly up-regulated in the infarcted hearts of HDC^−/−^ mice compared with controls (Fig. 3c).

### HDC-deficient-CD11b^+^ myeloid cells released proinflammatory cytokines and improved cardiac fibrogenesis post-MI

**Figure 4:**
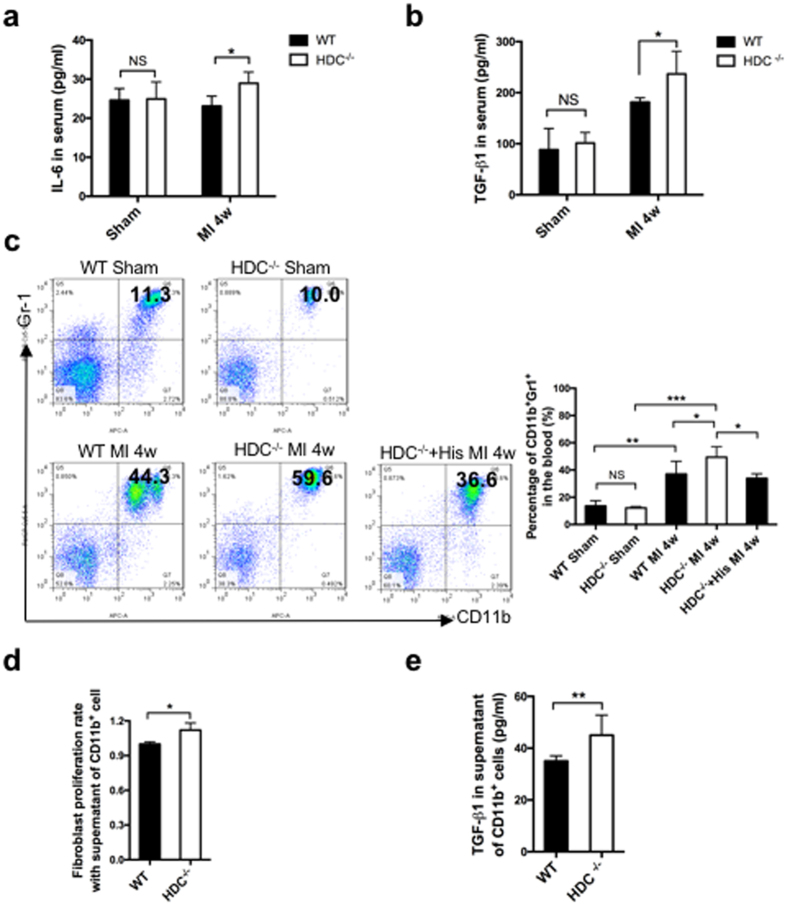
Histamine deficiency increased proinflammatory and profibrotic cytokines levels post-MI. (**a**,**b**) IL-6 (**a**) and TGF-β_1_ (**b**) levels in the serum of HDC^−/−^ mice and WT mice at 4 weeks post-MI were measured by ELISA (n = 6). (**c**) FACS data showed the percentage of CD11b^+^ myeloid cell numbers in the blood of HDC^−/−^ mice and WT mice at 4 weeks post-MI (n = 8). (**d**) The neonatal heart fibroblast proliferation rates were measured by the CCK-8 assay (n = 6). (**e**) TGF-β_1_ levels were measured by ELISA in the supernatant of CD11b^+^ myeloid cells (n = 6). NS, not statistically significant, *p < 0.05, **p < 0.01, ***p < 0.001.

Given that cardiac fibrosis could be associated with chronic inflammation, we hypothesized that histamine deficiency affected inflammation-associated cardiac fibrogenesis post-MI. As the data showed, the levels of the proinflammatory cytokine IL-6 and the profibrotic cytokine TGF-β_1_ in the serum significantly increased in HDC^−/−^ mice compared with WT controls at 4 weeks post-MI (Fig. 4a,b). Given that CD11b^+^ myeloid cells played a crucial role in the immune responses after MI, we performed a microarray study on CD11b^+^ myeloid cells isolated from the bone marrow of HDC^−/−^ and WT mice at 1 week and 4 weeks post-MI. The microarray data revealed the up-regulations of several proinflammatory and profibrotic cytokine genes in the CD11b^+^ myeloid cells of HDC^−/−^ mice, indicating that the persistent inflammation observed in HDC^−/−^ mice may be partially due to the HDC-deficient CD11b^+^ myeloid cells (Supplementary Fig. S4a,b). The FACS data also showed that the percentages of CD11b^+^Gr-1^+^ myeloid cells in the circulation significantly increased in HDC^−/−^ mice compared with WT controls post-MI. These data also showed that exogenous histamine treatment could reverse these changes (Fig. 4c). Furthermore, we performed an *in vitro* study using WT neonatal heart fibroblasts co-cultured with the supernatants of CD11b^+^ myeloid cells sorted from the bone marrow of either HDC^−/−^ or WT mice. The cell counting assay (CCK-8) results showed that the supernatants from HDC-deficient CD11b^+^ myeloid cells significantly enhanced fibroblast proliferation compared with those from WT control cells (Fig. 4d). The TGF-β_1_ levels were higher in the supernatants of HDC-deficient CD11b^+^ myeloid cells than in those of WT control cells (Fig. 4e). These results suggested that histamine deficiency enhanced inflammation-associated cardiac fibrogenesis in part by increasing the production of proinflammatory and profibrotic cytokines from bone marrow-derived CD11b^+^ myeloid cells post-MI.

### Histamine repressed fibroblast proliferation via a histamine receptor-dependent paracrine mechanism

**Figure 5:**
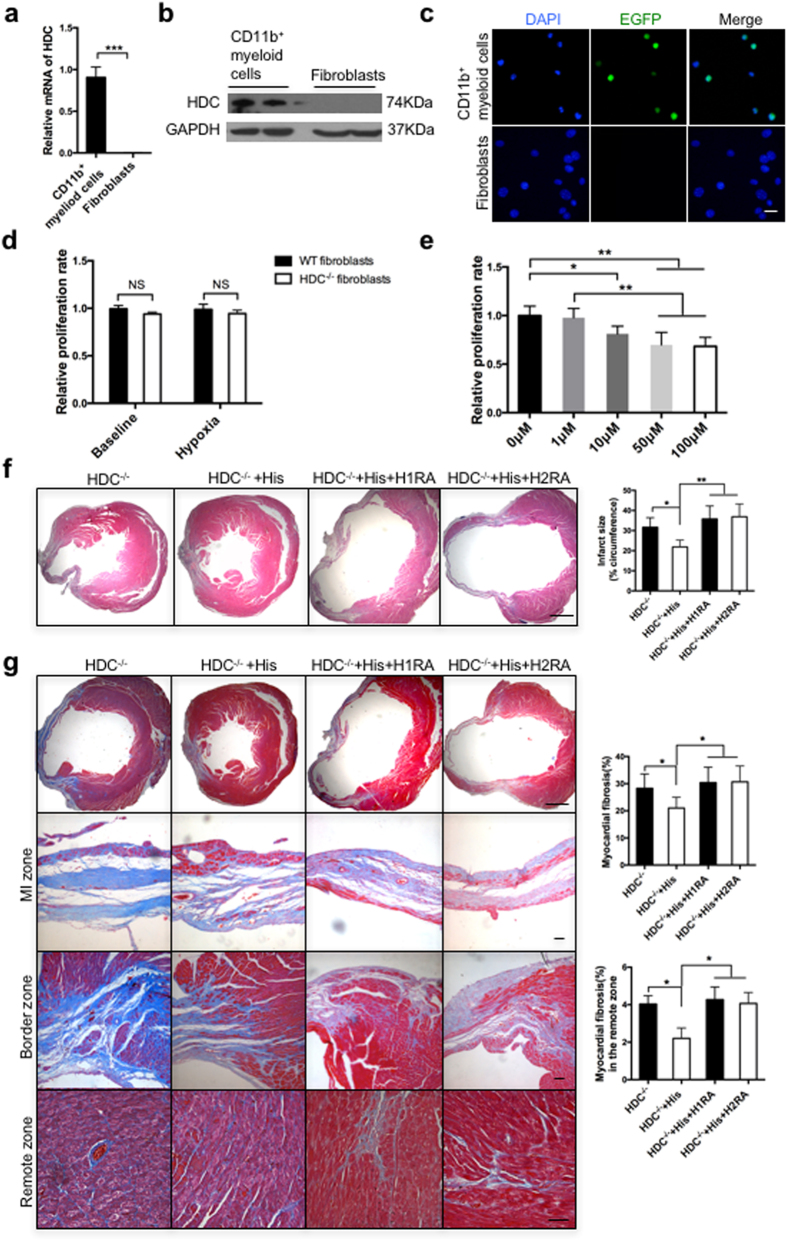
Histamine repressed fibroblast proliferation via a histamine receptor-dependent paracrine mechanism. (**a**,**b**) The qRT-PCR and western blotting data showed no expression of HDC in the heart fibroblasts (n = 3). (**c**) No EGFP expression in the heart fibroblasts of HDC-EGFP mice, as measured by fluorescence microscopy (scale bar = 25 μm). (**d**) The proliferation rates of neonatal heart fibroblasts of HDC^−/−^ and WT mice at baseline or in hypoxic conditions were measured by the CCK8 assay (n = 6). (**e**) Histamine (10^−5^ M~10^−4^ M) had an inhibitive effect on heart fibroblast proliferation (n = 3). (**f**) The results of H&E staining showed that the larger infarct size observed in HDC^−/−^ mice could be attenuated by histamine treatment. This effect could be blocked by H_1_R or H_2_R antagonists (scale bar = 500 μm; n = 6–8). (**g**) Masson trichrome staining showed that cardiac fibrosis decreased in histamine-treated HDC^−/−^ mice compared with HDC^−/−^ mice and that this effect was reversed by H_1_R or H_2_R antagonists (the top scale bar = 500 μm and other scale bars = 50 μm; n = 6–8). NS, not statistically significant, *p < 0.05, **p < 0.01, ***p < 0.001.

We next attempted to elucidate the mechanism underlying histamine-mediated repression of cardiac fibrogenesis. We hypothesized that HDC^+^CD11b^+^ myeloid cell-derived histamine inhibited heart fibroblast proliferation via a histamine receptor-dependent paracrine mechanism. To test this hypothesis, we first compared HDC expression levels in the heart fibroblasts and CD11b^+^ myeloid cells of WT mice by the qRT-PCR and western blotting assay. The results demonstrated higher expressions of the HDC mRNA and protein in CD11b^+^ myeloid cells compared with the heart fibroblasts (Fig. 5a,b). Second, the fluorescence microscopy data demonstrated that no EGFP expression was observed in the heart fibroblasts of HDC-EGFP mice compared with EGFP-expressing CD11b^+^myeloid cells (Fig. 5c). Furthermore, the CCK-8 data showed that there was no discernible difference in the proliferation rates of neonatal heart fibroblasts between HDC^−/−^ and WT mice at baseline or in hypoxic conditions (Fig. 5d). Although heart fibroblasts did not produce histamine, we were curious as to whether they were responsive to histamine. We observed that low concentrations of histamine (less than 10^−6^ M) had a mild effect on fibroblast proliferation, whereas higher doses of histamine (10^−5^ M~10^−4^ M) actually directly inhibited fibroblast proliferation (Fig. 5e). We next investigated H_1_ and H_2_ receptors (H_1_R and H_2_R) expressions in heart fibroblasts using immunofluorescence staining and western blotting (Supplementary Fig. S5a,b). We found that both H_1_ and H_2_ receptors were expressed in heart fibroblasts. Then, we utilized histamine receptor antagonists to determine which receptor conveyed the function of histamine during this process. We randomly divided HDC^−/−^ mice into the following four groups: an MI group, an MI + histamine group, an MI + histamine + H_1_R antagonist group, and an MI + histamine + H_2_R antagonist group. The histopathology and echocardiographic results revealed that the increases in cardiac diameter and volume and fibrosis that were observed in HDC^−/−^ mice with MI could be attenuated by histamine administration; however, pre-treatment with H_1_R or H_2_R antagonists abrogated the protective effects of exogenous histamine on MI-induced cardiac remodeling and heart failure in HDC^−/−^ mice (Fig. 5f,g and Supplementary Fig. S5c–h). To identify the appropriate time for the effects of histamine, exogenous histamine was administered into HDC^−/−^ mice during the first week, the fourth week, or the whole process after MI respectively (Supplementary Fig. S6a). Interestingly, we found that histamine injection in the first week exhibited the similar protective effects on cardiac function and fibrogenesis compared with 4-weeks histamine rescue (Supplementary Fig. S6b,c). Thus, these data indicated that histamine appeared to suppress cardiac fibrogenesis through H_1_R- and H_2_R-dependent signaling pathways.

### Histamine exerted protective effects on cardiac fibrosis through a STAT6-dependent signaling pathway

**Figure 6:**
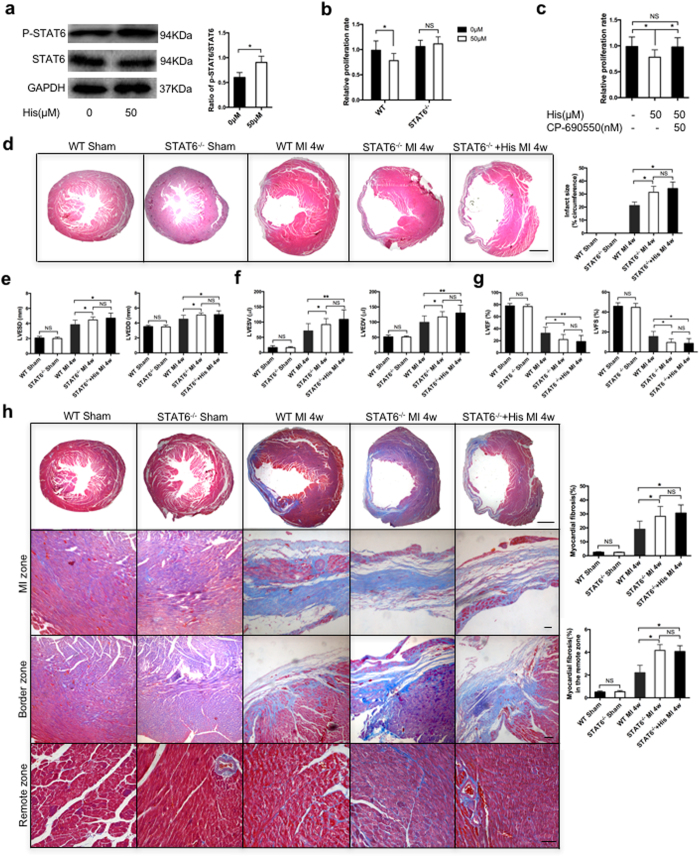
STAT6 signaling expression and function in histamine-medicated regulation of cardiac fibrosis post-MI. (**a**) Western blotting showed that exogenous histamine (10^−5^ M) could increase phosphorylated STAT6 expression in the heart fibroblasts of WT mice (n = 3). (**b**) Exogenous histamine (10^−5^ M) had no inhibitive effect on the heart fibroblasts of STAT6^−/−^ mice (n = 3). (**c**) The inhibitive effect of histamine on the proliferation of heart fibroblasts of WT mice could be blocked by CP-690550 (n = 3). (**d**) H&E staining showed that STAT6 knockout caused larger infarct sizes at 4 weeks post-MI (scale bar = 500 μm; n = 8–10). (**e–g**) Echocardiography showed that STAT6^−/−^ mice had worse cardiac remodeling and function post-MI than WT mice and that exogenous histamine had no beneficial effect on STAT6^−/−^ mice (n = 10–12). (**h**) Masson trichrome staining showed that cardiac fibrosis was significantly enhanced in STAT6^−/−^ mice compared with WT mice and that exogenous histamine had no beneficial effect on STAT6^−/−^ mice (the top scale bar = 500 μm and other scale bars = 50 μm; n = 6–8). NS, not statistically significant, *p < 0.05, **p < 0.01.

Our previous study reported that STAT6 gene expression was down-regulated in the CD11b^+^ myeloid cells of HDC^−/−^ tumor-bearing mice compared with the corresponding cells of WT mice^7^. Thus, we postulated that the STAT6-dependent signaling pathway may be involved in modulating the effects of histamine on cardiac fibrogenesis. First, our western blotting data demonstrated that phosphorylated STAT6 expression levels increased in the heart fibroblasts of WT mice treated with histamine (10^−5^ M) (Fig. 6a). Second, our CCK-8 data confirmed that histamine (10^−5^ M) did not inhibit the proliferation of heart fibroblasts isolated from STAT6^−/−^ mice (Fig. 6b). Using CP-690550, a selective JAK3/STAT6 inhibitor, we determined that the inhibitive effects of histamine on heart fibroblast proliferation in WT mice could also be abrogated (Fig. 6c). Finally, we used STAT6^−/−^ mice to validate whether the STAT6 signaling pathway was the intracellular signaling pathway that mediated the effects of histamine in MI-induced cardiac fibrogenesis. The histopathology and echocardiographic results showed that similar to HDC^−/−^ mice, STAT6^−/−^ mice displayed worse cardiac remodeling and function than WT mice post-MI, as these mice had larger LVEDDs, LVESDs, LVEDVs, and LVESVs (Fig. 6d–f) and lower LVEFs and LVFSs than their counterparts (Fig. 6g). The Masson trichrome staining and Picrosirius red staining demonstrated that the cardiac fibrosis rate was significantly enhanced in STAT6^−/−^ mice compared with WT mice (Fig. 6h and Supplementary Fig. S7). Interestingly, in contrast to its effects on HDC^−/−^ mice, exogenous histamine, which was injected intraperitoneally, had no beneficial effects on the more severe cardiac abnormalities of STAT6^−/−^ mice post-MI (Fig. 6d–h). Taken together, these data indicated that histamine exerted protective effects on cardiac remodeling at least in part through the STAT6 signaling pathway.

## Discussion

The results of this study revealed that histamine was strongly induced in a permanent occlusion model of MI and provided endogenous protection against MI-induced cardiac remodeling, at least in part, via the STAT6 signaling pathway. Here, we found that HDC-expressing CD11b^+^ myeloid cell numbers markedly increased in the circulation and infarcted hearts, from the early stage of MI to the late stage of cardiac remodeling. Histamine deficiency resulted in worse cardiac remodeling and dysfunction post-MI, because of increased early myocardial apoptosis^10^ and late cardiac fibrosis. Histamine treatment enhanced STAT6 phosphorylation in heart fibroblasts and inhibited cultured heart fibroblast proliferation. Furthermore, STAT6 knockout blocked the protective effect of exogenous histamine on MI-induced cardiac fibrosis and remodeling. Thus, our data indicated that the histamine-STAT6 axis may be a potential target for preventing MI-induced cardiac remodeling and heart failure.

The general assumption suggested in previous studies has been that mast cells could release large amounts of histamine in response to stimuli^15^,^16^,^17^. However, CD11b^+^ myeloid cells, particularly CD11b^+^Gr-1^+^ myeloid cells within the bone marrow and spleen, were identified as the predominant HDC-expressing cellular pools within HDC-EGFP transgenic mice^7^. In this study, we found that circulating histamine levels were significantly up-regulated on day 3 post-MI and subsequently exhibited a gradual decline up to 4 weeks post-MI. The presence of EGFP^+^CD11b^+^ myeloid cells in the circulation and infarcted hearts of HDC-EGFP mice coincided with the above changes in histamine levels, confirming that these cells were the source of endogenous histamine during MI.

In the setting of MI, the extracellular matrix can undergo a series of dynamic changes leading to favorable ventricular remodeling and functional adaptation. These changes can be arbitrarily divided into the following three phases: the early injury response phase (from 0 to 48 hours post-MI), the proliferation phase (between 2 and 5 days post-MI), and the late maturation phase (≈1 month post-MI)^18^. However, sustained and excessive interstitial fibrous tissue deposition causes adverse cardiac remodeling and impairs cardiac function^18^. Many factors have been implicated in MI-induced cardiac remodeling, including the TGF-β/Smad signaling pathway, reactive oxygen species^19^, matrix metalloproteinases^20^, and the renin angiotensin aldosterone system^21^. The role of histamine in fibrosis has been investigated in other pathologic conditions^22^,^23^,^24^; however, the variability in the models used and the concentrations of histamine employed have precluded sharp conclusions. In this study, we demonstrated that histamine deficiency promoted cardiac fibrogenesis and heart dysfunction *in vivo*. On one hand, increased levels of the profibrotic cytokine TGF-β_1_ were detected in HDC^−/−^ mice, suggesting that histamine was likely to indirectly influence cardiac fibrosis by coupling with classical profibrotic signaling pathways. On the other hand, CD11b^+^ myeloid cell-derived histamine had a direct inhibitive effect on heart fibroblast proliferation. Thus, we believe that histamine may have both direct and indirect ways of modulating cardiac remodeling post-MI.

Cardiac repair post-MI is dependent on proper mobilization of the immune system, which functions to clean dead cells and matrix debris and to activate repair programs^25^. However, it is well established that timely repression of inflammatory signals is necessary to ensure optimal healing in the infarcted hearts and to prevent the development of adverse remodeling^26^,^27^,^28^. Histamine deficiency not only repressed macrophage differentiation but also caused impaired macrophage infiltration and delayed dead myocardial cells clearance^7^,^10^. These may act as a sustained alarm to further recruit immune cells to release proinflammatory cytokines. Our results showed that CD11b^+^Gr1^+^ myeloid cells were more abundant in the circulation of HDC^−/−^ mice than in the circulation of WT control mice and were associated with increased proinflammatory cytokines production during cardiac remodeling development. A persistent inflammatory microenvironment in the infarcted hearts of HDC^−/−^ mice may be one of the mechanisms underlying adverse cardiac remodeling.

Intracellular signaling pathways downstream of histamine in heart fibroblasts have not been well studied^29^,^30^. We previously demonstrated that STAT6 mRNA expression levels decreased in CD11b^+^ myeloid cells from colon tumor-bearing HDC^−/−^ mice compared with WT control mice^7^. The role of STAT6 activation in fibrosis has been investigated in other organs, such as the lung, skin, and kidney, using STAT6^−/−^ mice^31^,^32^,^33^. In this study, we uncovered three lines of evidences supporting the hypothesis that the STAT6 signaling pathway was one of the major intracellular histamine pathways in heart fibroblasts. First, we found that exogenous histamine enhanced STAT6 phosphorylation levels in heart fibroblasts. Second, we found that treatment with CP-690550, a JAK3/STAT6 signaling selective antagonist, blocked the effects of histamine on heart fibroblast proliferation. Third, we observed increased cardiac fibrosis in the injured hearts of STAT6^−/−^ mice, results similar to those observed in HDC^−/−^ mice, whose phenotype could not be rescued by treatment with exogenous histamine. Taken together, these data suggested that CD11b^+^ myeloid cell-derived histamine exerted its protective effect on cardiac fibrosis possibly via a STAT6-dependent signaling pathway. Additional studies are needed to delineate the precise mechanisms underlying the histamine-mediated crosstalk between CD11b^+^ myeloid cells and heart fibroblasts.

Some questions remain unclear regarding the relationships between histamine (or histamine receptor antagonists) and MI. Previous studies reported that a better prognosis was observed in MI patients with higher serum IgE levels, a phenomenon that may be attributed to increased histamine release from mast cells^34^. However, other studies showed that histamine H_2_ receptor antagonists could improve cardiac function in chronic heart failure patients, as well as in chronic heart failure mice induced by transverse aortic constriction model^23^,^35^,^36^. These results may be attributed to decreases in both blood pressure and heart rate, changes similar to those elicited by beta-adrenoreceptor blockers^35^. Although both transverse aortic constriction model and MI model profoundly influence cardiac remodeling, different pathological cellular and molecular events are involved in these two different heart failure models. Moreover, there are four types of histamine receptors^11^,^37^, and both H_1_R and H_2_R are highly expressed on the cardiomyocytes, vascular cells, and fibroblasts of the heart^38^. Compared with the data from previous studies, our data indicated that a blockade of H_1_R- and H_2_R-dependent signaling secondary to histamine deficiency resulted in worse cardiomyocyte apoptosis, cardiac fibrogenesis, and dysfunction in mice following MI. Interestingly, our data indicated that the protective effects of histamine in MI-induced cardiac remodeling depended on the appropriate time and concentration to use it. Thus, elucidating the different signaling pathways involved in the effects of different histamine receptors on various cells may play an integral role in understanding the diverse effects of histamine. These controversial conclusions highlight the complexity of histamine function in cardiovascular diseases. Thus, additional studies are needed to clarify the roles of histamine and its receptors in MI, as well as other models of heart failure.

In conclusion, we presented several lines of evidences indicating that endogenous histamine plays a protective role in the evolution from MI to heart failure. Additional studies will be needed to address whether the histamine-STAT6 axis could be a useful therapeutic target for the treatment of MI-induced heart failure.

## Methods

### Patient characteristics and serum histamine determination by ELISA

Serum samples were collected from heart failure patients who were admitted to Zhongshan Hospital, Fudan University. The MI histories of patients with New York Heart Association (NYHA) class IV were confirmed by angiography. Serum samples from the healthy control subjects were collected at the TongRen Hospital Affiliated with Shanghai Jiao Tong University. The investigations complied with the ethics guidelines of the 1975 Declaration of Helsinki and were approved by the review boards on human subject research in our institutions (Zhongshan Hospital, Fudan University and TongRen Hospital Affiliated with Shanghai Jiao Tong University). Informed consent was obtained from all participants. Serum histamine concentrations were determined using a Histamine ELISA Kit (EA213/96, Eagle Biosciences), and were detected by a microplate reader (SpectraMax M5).

### Experimental mice

HDC-EGFP mice (C57BL/6 background) and HDC^−/−^ mice (Balb/C background) were generously provided by Professor Timothy C. Wang from Columbia University^7^. STAT6^−/−^ mice (Balb/C background) were purchased from the Model Animal Research Center of Nanjing University^39^. Balb/C mice and C57BL/6 WT mice were purchased from the Department of Laboratory Animal Science, Fudan University, to serve as background controls. All mice were housed under specific-pathogen-free conditions with a 12/12 hours day/night cycle. Anesthesia was administered in the form of 1.0–2.0% isoflurane gas, and the mice were euthanized by cervical dislocation. The mice received piritramide (10 mg/kg body weight) perioperatively and tramadolhydrochloride (2.5 mg/100 ml drinking water) postoperatively for the first seven days after surgery. Our study conformed to the Guide for the Care and Use of Laboratory Animals published by the US National Institutes of Health (NIH Publication, 8th Edition, 2011). The protocol was approved by the Committee on the Ethics of Animal Experiments of Fudan University (approval reference number: SY2014.2.001.002).

### MI-induced heart failure model

MI model was induced by ligating the LAD in mice, as previously described^40^. Briefly, the mice were anesthetized and intubated with a 22-G intravenous catheter, and then mechanically ventilated with 1.0–2.0% isoflurane gas using a rodent respirator. Left thoracotomy was performed at the fourth intercostal space, and the LAD was identified and ligated at 2–3 mm from the tip of the left auricle using an 8–0 silk suture. Ligation success was confirmed when the anterior wall of the left ventricle turned pale. Sham-operated mice underwent the same surgical procedures; however, the suture placed under the LAD was not tied. The chest cavity was closed with a continuous 6–0 prolene suture, and the animal was placed in a cage on a heating pad. Histamine (4 mg/kg/d), an H_1_R antagonist (pyrilamine, 10 mg/kg/d) and an H_2_R antagonist (cimetidine, 10 mg/kg/d) were administered intraperitoneally daily beginning 3 days before surgery and continuing until 4 weeks after MI surgery. The physical conditions of the animals were evaluated two times per day. The causes of death after MI were mainly heart rupture during the first week and severe heart failure during the late stages post-MI.

### Echocardiography

Transthoracic echocardiography was performed 4 weeks after LAD ligation using a Vevo770 imaging system (VisualSonics, Inc.). The following parameters were quantified by digitally recorded two-dimensional short-axis M-mode tracings at the papillary muscle level: LVESD, LVEDD, LVESV, LVEDV, LVEF, and LVFS.

### Histology and immunofluorescence

WT, HDC^−/−^, and STAT6^−/−^ mouse hearts were fixed with 10% formalin for paraffin embedding. H&E staining and Masson staining were subsequently performed. Picro-Sirius Red staining (ab150681, Abcam) was also used for detecting fibrosis and viewed using standard light microscopy. For immunofluorescence staining, HDC-EGFP mouse hearts were fixed with 4% paraformaldehyde for 24 hours, followed by 30% sucrose overnight. Primary antibodies to CD11b (Abcam), H1R (sc-20633 Santa Cruz Biotechnology) and H2R (sc-19773 Santa Cruz Biotechnology) were used to perform immunofluorescence staining on frozen tissue sections, according to the manufacturer’s instructions.

### Flow cytometry analysis

The mice were euthanized at 4 weeks after MI. Their blood, spleens, bone marrow and infarcted hearts were collected and made into single-cell suspensions for flow cytometry analysis, as previously described^10^. The cells were stained with a mixture of antibodies (anti-CD11b-APC and anti-Gr-1-PerCP-Cy5.5 BD Biosciences). The data were acquired using an LSRII flow cytometer (BD Biosciences) and were analyzed with FlowJo7 software (Tree Star, Inc.).

### ELISA assay

Serum samples were collected from mice euthanized at 4 weeks post-MI. PICP, PIIINP, IL-6, and TGF-β_1_ expressions were measured with the respective ELISA kits (Beijing 4A Biotech Co., Ltd, China).

### Measurement of cell apoptosis

WT and HDC^−/−^ mouse hearts were fixed with 10% formalin for paraffin embedding, and 0.5-μm sections were prepared for staining. Apoptotic cells were detected by terminal deoxynucleotidyl transferase-mediated dUTP nick-end labeling (TUNEL), according to the manufacturer’s recommendations (Biotech Well, China).

### Cell culture and CCK-8 assay

CD11b^+^ myeloid cells from the bone marrow of WT and HDC^−/−^ mice were sorted by magnetic beads and cultured as previously described^10^. Anesthesia was administered in the form of 1.0–2.0% isoflurane gas, and the mice were euthanized by cervical dislocation. Heart fibroblasts were isolated from 3-day-old mice by enzymatic digestion. The fibroblast proliferation assay was performed using the CCK-8 assay (Biotech Well, China), as previously described^41^. Briefly, the fibroblasts were incubated in 96-well plates at 37 °C under normoxic conditions (5% CO2 and 20% O2), and assays were performed 24 hours after plating by adding 100 μl of fresh medium and 10 μl of the CCK-8 solution to the wells for another 2 hours at 37 °C. The OD at 450 nm was measured. The results were representative of more than three individual experiments.

### Western blotting

Following the appropriate treatments, cultured cells or infarcted heart tissue were lysed with RIPA lysis buffer (Beyotime Biotechnology, China) for 30 min, followed by centrifugation at 14,000× g for 30 min. The protein concentration of each sample was quantified via BCA protein assay (Biotech Well, China). Then, equal amounts of proteins were electrophoresed on a 6% to 15% gradient gel by sodium dodecyl sulfate-polyacrylamide gel electrophoresis and transferred to polyvinylidene difluoride membranes. The membranes were blocked with 5% BSA in Tris-buffered saline and 0.2% Tween (TBST) at room temperature for 1 hour and then incubated overnight at 4 °C with the following specific primary antibodies: glyceraldehyde-3-phosphate dehydrogenase (GAPDH) (ab8245, Abcam), β-actin (ab8227, Abcam), STAT6 (ab44718, Abcam), p-STAT6 (ab54461, Abcam), HDC (ab37291, Abcam), H1R (sc-20633, Santa Cruz Biotechnology) and H2R (sc-19773, Santa Cruz Biotechnology). The blots were then washed three times with TBST and incubated with a horseradish peroxidase-conjugated secondary antibody (Biotech Well, China) for 45–60 min at room temperature. The expression signals were detected with an enhanced chemilumi-nescence reagent (Dako, Denmark) after the membranes had been washed with TBST (10 min × 3). Protein expression levels were quantified by scanning densitometries and standardized by GAPDH or β-actin.

### Quantitative reverse transcription-PCR (qRT-PCR)

Total RNA was extracted using TRIzol reagent (Invitrogen, MA, USA). Quantitative gene expression analyses for HDC, collagen I, collagen III, MMP-2, and MMP-9 mRNA were performed using SYBR Premix Ex Taq (Takara, Japan) with an Applied Biosystems Prism 7500 sequence detection system. The results were standardized to GAPDH control values. The average of three independent analyses for each gene was calculated. The PCR primers were designed by Sangon Biotech (Table 2).

### Statistical analysis

The data were expressed as the mean value ± standard deviation. Survival rate was determined using the Kaplan-Meier method. The difference between survival curves was determined using the log-rank test. Comparisons between the two groups were assessed by *t-*test. For comparisons between more than two groups, we used one-way ANOVA if there was one independent variable and two-way ANOVA if there were two independent variables. Bonferroni correction was performed for multiple comparisons. In all analyses, *p* < 0.05 was considered statistically significant.

## Additional Information

**How to cite this article**: Chen, J. *et al*. Aggravated myocardial infarction-induced cardiac remodeling and heart failure in histamine-deficient mice. *Sci. Rep.* **7**, 44007; doi: 10.1038/srep44007 (2017).

**Publisher's note:** Springer Nature remains neutral with regard to jurisdictional claims in published maps and institutional affiliations.

## Supplementary Material

Supplementary Information

## Figures and Tables

**Table 1:** Echocardiographic results for the WT, HDC^−/−^, and STAT6^−/−^ mice at baseline.

	WT Sham	HDC^−/−^ Sham	STAT6^−/−^ Sham
LVEF (%)	76.82 ± 4.92	73.08 ± 8.10	75.75 ± 4.38
LVFS (%)	44.77 ± 4.35	42.30 ± 6.75	44.66 ± 4.97
LVESD (mm)	2.06 ± 0.32	2.11 ± 0.30	2.00 ± 0.28
LVEDD (mm)	3.52 ± 0.22	3.65 ± 0.11	3.48 ± 0.31
LVESV (μl)	16.53 ± 3.00	16.21 ± 3.05	16.14 ± 2.64
LVEDV (μl)	52.62 ± 4.05	58.86 ± 5.47	52.25 ± 4.75
LVDAW (mm)	0.85 ± 0.13	0.83 ± 0.12	0.81 ± 0.09
LVSAW (mm)	1.24 ± 0.12	1.20 ± 0.14	1.19 ± 0.11

LVEF: left ventricular ejection fraction; LVFS: left ventricular fractional shortening; LVESD: left ventricular end-systolic diameter; LVEDD: left ventricular end-diastolic diameter; LVESV: left ventricular end-systolic volume; LVEDV: left ventricular end-diastolic volume; LVDAW: left ventricular diastolic anterior wall thickness; LVSAW: left ventricular systolic anterior wall thickness. n = 6.

**Table 2:** Primers Used for qRT-PCR.

Gene	Forward primer	Reverse Primer
GAPDH	GACATCAAGAAGGTGGTGAAGCAG	ATACCAGGAAATGAGCTTGACAAA
HDC	TTAGTCTTTGGGTGTTCCTGGTCA	CCCTGTTGCTTGTCTTCCTCAATA
Collagen I	TTCTTCTGGCAAAGACGGAC	CGGCCACCATCTTGAGACTT
Collagen III	AAAGGGGCTGGAAAGTGAGG	AGCACCATCAGTTGTCCCTG
MMP-2	TGGAATGCCATCCCTGATAACC	CAGCCCAGCCAGTCTGATTTGA
MMP-9	CGTCATTCGCGTGGATAAGGAG	CCTGGTTCACCTCATGGTCCAC
