# Supplementary material for: Aggravated myocardial infarction-induced cardiac remodeling and heart failure in histamine-deficient mice
Source: Sci Rep. 2017 Mar 8;7:44007. doi: 10.1038/srep44007 (PMC5341031; doi:10.1038/srep44007)
Supplement: Supplementary Information [file srep44007-s1.pdf]

Aggravated myocardial infarction-induced cardiac remodeling and heart failure in histamine-deficient mice

Jinmiao Chen[1,2,†], Tao Hong[1,2,†], Suling Ding[1], Long Deng[1,2], Mieradilijiang Abudupataer[1,2], Weiwei Zhang[1], Minghong Tong[3], Jianguo Jia[1], Hui Gong[1], Yunzeng Zou[1], Timothy C. Wang[4], Junbo Ge[1*], Xiangdong Yang[1*]

1.Shanghai Institute of Cardiovascular Diseases, Zhongshan Hospital, and Institutes of Biomedical Sciences, Fudan University, Shanghai, 200032, China

2.Department of Cardiac Surgery, Zhongshan Hospital, Fudan University, Shanghai, 200032, China

3.Department of Clinical Medicine, TongRen Hospital Affiliated with Shanghai Jiao Tong University, Shanghai, 200000, China

4.Department of Medicine and Irving Cancer Research Center, Columbia University, New York, NY 10032, USA.

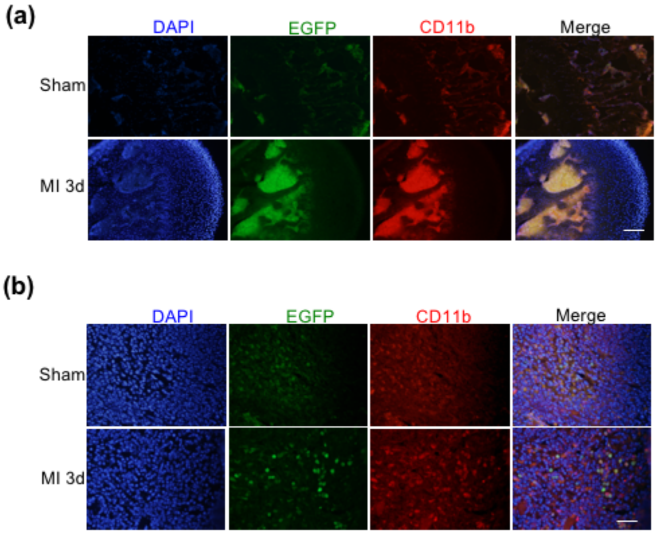

Figure S1. EGFP$^+$CD11b$^+$ myeloid cell numbers markedly increased in the bone marrow and spleens of HDC-EGFP mice at 3 days post-MI. (**a, b**) Representative images of the bone marrow (**a**) and spleens (**b**) of HDC-EGFP mice at 3 days post-MI (scale bar=50μm; n=5).

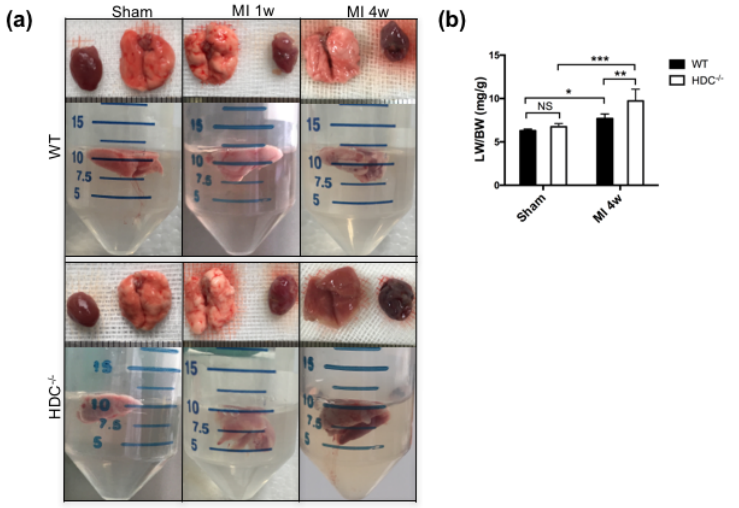

Figure S2. Histamine deficiency promoted the pulmonary edema associated with worse cardiac dysfunction after MI. (**a**) Representative images of the lungs in WT and $HDC^{-/-}$ mice at 1 week and 4 weeks post-MI. (**b**) Lung weights were higher in $HDC^{-/-}$ mice than WT mice at 4 weeks post-MI (n=8-10). NS, not statistically significant, *$p<0.05$, **$p<0.01$, ***$p<0.001$.

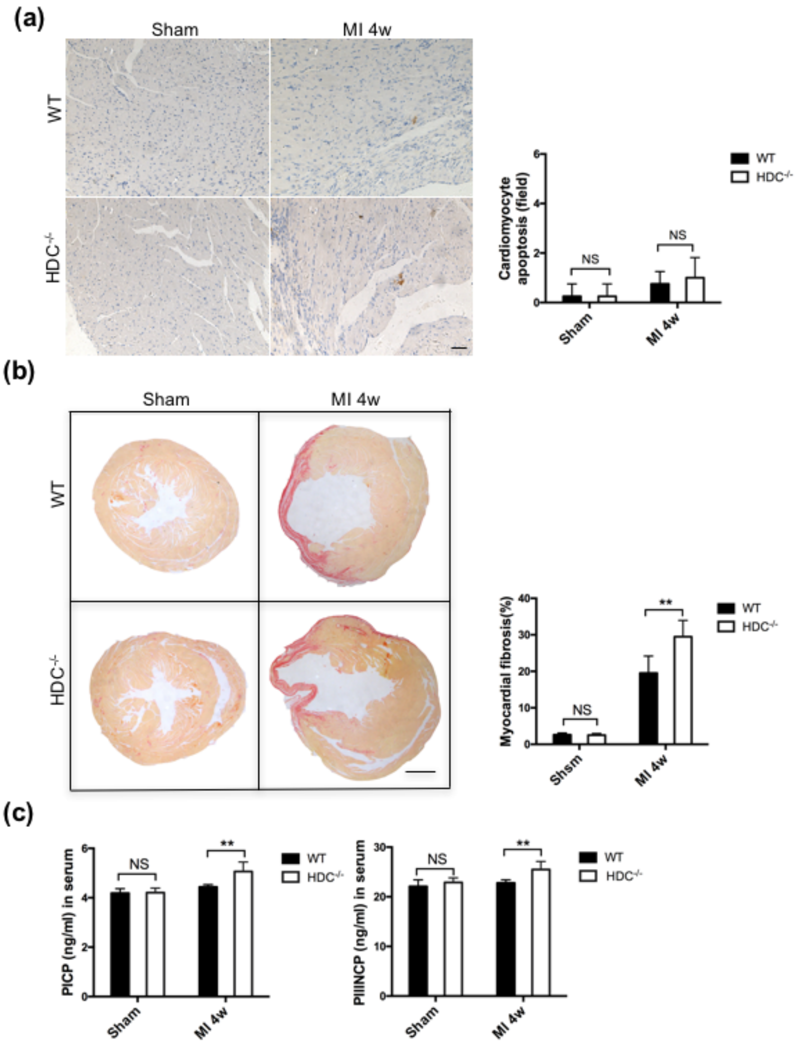

Figure S3. Histamine deficiency increased fibrosis in the infarcted hearts. (**a**) TUNEL staining showed no significant difference in the numbers of apoptotic cardiomyocytes between WT and HDC$^{-/-}$ mice at 4 weeks post-MI (scale bar=50μm; n=5). (**b**) The cardiac fibrosis rates in HDC$^{-/-}$ mice compared with WT controls were measured by the Picro-Sirius Red staining (the scale bar=500 μm; n=8-10). (**c**) PICP and PIIINP levels in the serum at 4 weeks post-MI were measured by ELISA assays (n=6-8). NS, not statistically significant, **p<0.01.

(a)

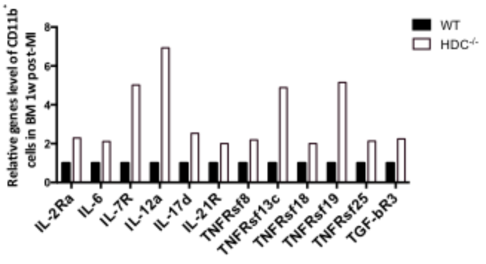

(b)

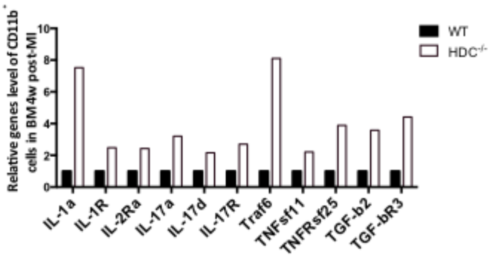

Figure S4. Up-regulations of several pro-inflammatory and pro-fibrotic cytokines in CD11b$^+$ myeloid cells isolated from the bone marrow of HDC$^{-/-}$ mice compared with WT control mice at 1week (**a**) and 4 weeks (**b**) post-MI. The microarray assay was performed on the bone marrow-derived CD11b$^+$ myeloid cells that were pooled by five mice in each group.

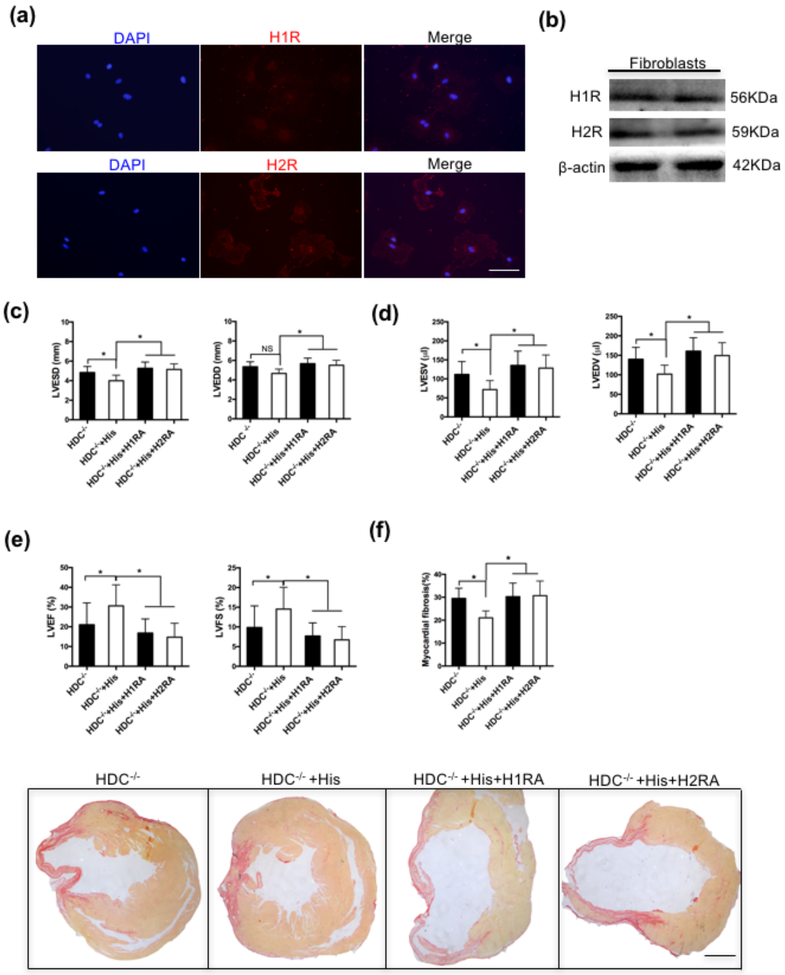

Figure S5. Histamine repressed cardiac fibrosis through H_1R- and H_2R-dependent pathways. (**a, b**) H_1 and H_2 receptors could be detected in the heart fibroblasts by (**a**) immunofluorescence staining (scale bar=50μm) and (**b**) western blotting. (**c-e**), The echocardiographic data demonstrated that worse cardiac remodeling and dysfunction in HDC$^{-/-}$ mice at 4 weeks post-MI. The protection of histamine injection could be blocked by H_1R or H_2R antagonists (n=8-10). (**f**) The Picro-Sirius Red staining showed that cardiac fibrosis decreased in histamine-treated HDC$^{-/-}$ mice compared with HDC$^{-/-}$ mice and that the effects of histamine were reversed by H_1R or H_2R antagonists (the scale bar=500 μm; n=6-8). NS, not statistically significant, *p<0.05, **p<0.01.

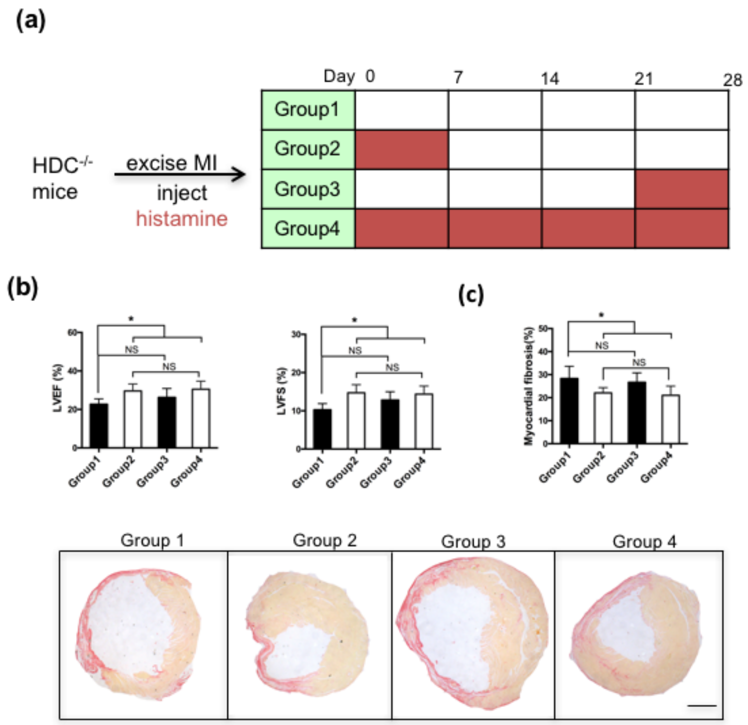

Figure S6. Histamine curative study. (**a**) Schematic of histamine curative study. (**b**) Heart functions were examined by echocardiographic assay (n=8). (**c**) Cardiac fibrosis rates were analyzed by the Picro-Sirius Red staining (the scale bar=500 µm; n=8). NS, not statistically significant, *$p<0.05$.

(a)

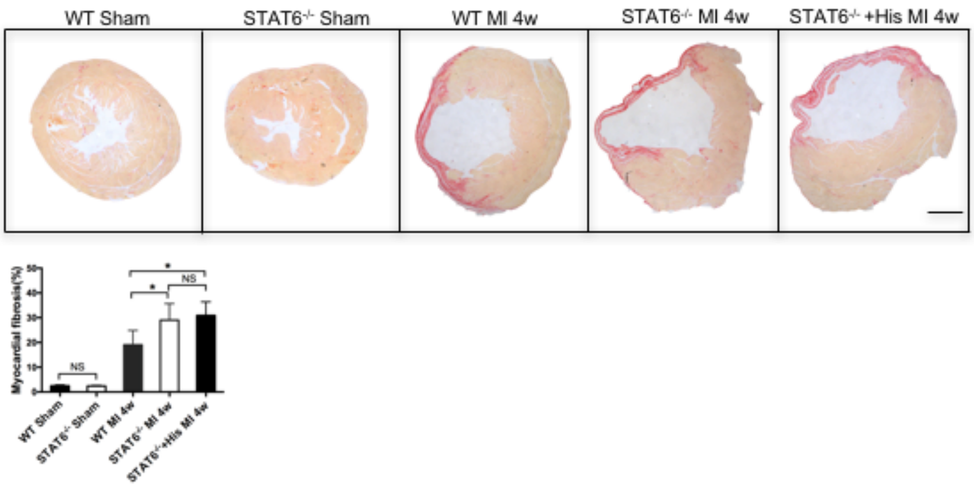

Figure S7. The Picro-Sirius Red trichrome staining showed that cardiac fibrosis rate was higher in STAT6$^{-/-}$ mice than WT mice at 4 weeks post-MI, and that exogenous histamine had no beneficial effect on STAT6$^{-/-}$ mice (the scale bar=500 μm; n=6-8). NS, not statistically significant, *$p<0.05$.
